# Phenomenological modelling of non-volatile memory threshold voltage shift induced by nonlinear ionization with a femtosecond laser

**DOI:** 10.1038/s41598-019-43344-x

**Published:** 2019-05-14

**Authors:** Philippe Chiquet, Maxime Chambonneau, Vincenzo Della Marca, Jérémy Postel-Pellerin, Pierre Canet, Sarra Souiki-Figuigui, Guillaume Idda, Jean-Michel Portal, David Grojo

**Affiliations:** 1 Aix-Marseille University, CNRS, IM2NP, F-13397 Marseille, France; 2 Aix-Marseille University, CNRS, LP3, F-13288 Marseille, France

**Keywords:** Electronics, photonics and device physics, Optics and photonics

## Abstract

The behaviour of semiconductor materials and devices subjected to femtosecond laser irradiation has been under scrutiny, for many reasons, during the last decade. In particular, recent works have shown that the specific functionality and/or geometry of semiconductor devices, among which non-volatile memory (NVM) devices hold a special place, could be used to improve the knowledge about ultrafast laser-semiconductor interactions. So far, such an approach has been applied to draw conclusions about the spatio-temporal properties of laser propagation in bulk materials. Here, by comparing the evolution of the electrical characteristics of Flash cells under the cumulative effect of repeated femtosecond laser pulses with first-order physical considerations and TCAD (Technology Computer Aided Design) simulations, we clearly establish the role of the carriers created by nonlinear ionization on the functionality of the structures. The complete electrical analysis informs indirectly on the energy of the laser-produced free-carriers which, to date, was almost inaccessible by an experimental method applicable to the bulk of a material. Establishing the link between the carrier energy and laser parameters is of major importance to improve the comprehension of the nonlinear ionization mechanisms associated to intense laser-semiconductor interactions and applied in various fields from microelectronics to laser micromachining.

## Introduction

**Figure 1:**
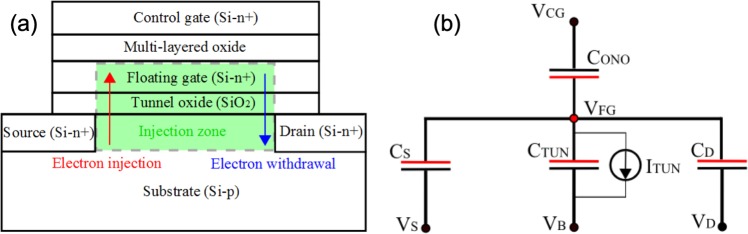
(**a**) Schematic representation of a standard Flash memory cell. (**b**) Electrical equivalent model of a standard Flash memory cell displaying the terminals of the floating gate transistor (control gate – CG, source – S, bulk – B, drain – D).

One of the important reasons for which the laser/semiconductor interaction has been investigated during the last two decades lies in the increasing importance of single-event effects (SEE) occurring in nowadays electronic circuits. In particular, single-event upsets (SEU), defined as the flips of a logical state or transient changes of an electrical parameter, occur when said circuits are exposed to energetic particles in radiative environments. Such systems should ideally be tested with the help of particle accelerators, but this solution remains both costly and impractical. In order to emulate the effect of energetic particles such as heavy ions while avoiding the associated radiation damage that comes with it, an alternative solution consists in generating charge carriers in circuits or individual components by irradiating them with laser beams^1,2^. The development of nonlinear optics has shown that the spatial and temporal evolutions of the density of carriers generated through laser irradiation is ruled by equations dependent on *I*^*n*^, where *I* is the intensity of the laser beam at a given wavelength and *n* is the number of absorbed photons required to generate a single free electron-hole pair in the semiconductor^3–8^. As a direct consequence, carrier generation in (nonlinear) modes such as *n* > 1 is characterized by a carrier density which is spatially confined near the focus of the laser beam, allowing to target specific locations in a component or a circuit as long as his characteristic dimensions remain larger than the laser spot size. Since the feasibility of through-wafer two-photon absorption resulting from femtosecond laser irradiation has been demonstrated^9,10^, transient and charge collection measurements performed on semiconductor devices such as bipolar transistors, Fin Field-effect transistors (FinFETs), and diodes have been compared to numerical simulations in order to be able to emulate and further understand SEEs thanks to this method^11–17^. Our recent works have shown that the peculiarities of other semiconductor devices, such as Flash memory cells, can be used as a way to characterize the carriers generated by femtosecond laser irradiation in semiconductors^18,19^. Flash memory cells can be described, as depicted in Fig. 1, as regular Metal-Oxide-Semiconductor (MOS) transistors on which a multi-layered oxide (generally a SiO_2_/Si_3_N_4_/SiO_2_ tri-layer) and an additional control gate (CG) have been added. The threshold voltage *V*_*t*_ of the device can be modulated according to the amount of charge *Q*_*FG*_ stored in the floating gate (FG), and both electrical quantities can be linked by the following relationship:

1 $$Vt=V{t}_{0}-\frac{{Q}_{FG}}{{C}_{ONO}}$$

where *Vt*_0_ and *C*_*ONO*_ are respectively the  neutral threshold voltage (i.e. the particular *V*_*t*_ value for which *Q*_*FG*_ = 0) and the capacitance of the FG/ONO/CG stack. According to the sign of *Q*_*FG*_, the floating gate transistor can be driven into two distinct and stable logical states (“erased” when *Q*_*FG*_ > 0 and “programmed” when *Q*_*FG*_ < 0). The floating gate charge variation necessary for the device to switch from one state to the other is assured when electron transit through the tunnel oxide is enabled by the application of suitable electric signals to the various terminals of the transistor. Flash cells are generally programmed via injection of hot electrons into the floating gate, and erased when electrons are withdrawn from the floating gate through Fowler-Nordheim conduction^20–25^.

We have recently shown that femtosecond laser irradiation of initially programmed and erased Flash cells could provoke their progressive shifting towards the opposite electrical state^18,19^. As the biasing conditions of the cells were neither compatible with hot carrier injection nor with Fowler-Nordheim conduction during the laser shots, charge carrier transit through the injection zone, for laser energies below the threshold for irreversible device degradation, was attributed to the tunneling of energetic carriers created in the substrate and the floating gate.

The present article completing these previous studies deals with the modelling of the ultrafast laser-induced shift of the threshold voltage. By varying the laser intensity, it is found that the laser-induced programming speed of Flash memories can be controlled. Additionally, the asymptotical logical state of the irradiated devices can be adjusted by applying a bias voltage on the control gate. A good agreement is found between the experiments and first-order physical considerations as well as Technology Computer Aided Design (TCAD) simulations, which both enable us to access to the laser-produced free carrier dynamics. The whole set of results enhance the global understanding of the femtosecond laser-transistor interaction and demonstrates the possibility to impact single isolated Flash memories with light.

## Results and Discussion

### Electrical behaviour of the injection zone during femtosecond laser irradiation

**Figure 2:**
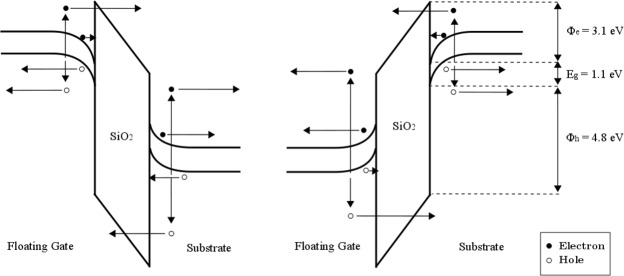
Band diagram of the injection zone of the Flash cell (i.e. electron energy as a function of vertical position in said zone) represented for both possible directions of the vertical electric field. Vertical arrows indicate the creation of electron-hole pairs in both electrodes under laser irradiation, while the horizontal arrows depict the motion of free electrons (plain circles) and holes (empty circles), whether laser-generated or not, under the influence of the electric field. The physical quantities *E*_*g*_, *ϕ*_*e*_ and *ϕ*_*h*_ are respectively the silicon bandgap and the Si-SiO_2_ barrier heights for electrons and holes.

Backside femtosecond laser irradiation, after altering the p-doped silicon substrate of the Flash cells, reaches the n-doped silicon floating gate with a diminished intensity after successive reflections on both silicon/oxide interfaces. During a given laser pulse, the strength and orientation of the vertical electric field reigning in the injection zone are determined by *Q*_*FG*_ and the voltages applied to the terminals of the transistor, as depicted in Fig. 2. Due to the nature of the experiments led in this study, and in particular the fact that no measurement configuration will involve a non-zero value of *V*_*D*_, the norm of the vertical oxide field can be considered as constant in the longitudinal direction (i.e., across the transistor channel located between the source and the drain).

Electron-hole pairs are generated in both electrodes with two-photon ionization, which is the dominant mechanism for carrier production in silicon at the considered wavelength of 1300 nm^9^. According to the orientation of the field, as shown in Fig. 2, electrons and holes, whether they result from laser irradiation or were already present in the silicon at thermodynamic equilibrium, can flow into (or get extracted from) the floating gate through the tunnel oxide with a non-zero probability.

This basic model, in the light of which the subsequent experimental results will be interpreted, also relies on a few assumptions backed up by physical considerations or results discussed in our previous studies^18,19^. More specifically, it will be considered that femtosecond laser irradiation will not affect the electrical properties of the tunnel oxide because of the high value of the SiO_2_ bandgap (around 9 eV) that would require ten-photon absorption to come into play for laser energy deposition at 1300-nm wavelength. Briefly, the theoretical scenario describing the behaviour of Flash memory device irradiated by ultrashort laser pulses relies on the tunneling of free electrons through the oxide layer of the cell before the electron-hole pairs potentially recombine in a few ns^26^. Depending on the migration of these free electrons produced by two-photon ionization, the net charge of the floating gate is changed by this way. One must emphasize that the employed low repetition rate of 1 kHz ensures no thermal/electron accumulation on a pulse-to-pulse basis. In other words, the electron-hole pairs created by one pulse have recombined when the following pulse irradiates the cell. It can thus be concluded that the only consequence of individual laser shots, after thermodynamic equilibrium is reached again, lies in a change of the floating gate charge and the potential distribution in the vertical direction of the injection zone. In our previous works^18,19^ no damage of the tunnel oxide (surface or volume) has been observed, even at laser energies much higher than the ones employed in this study. This can indicate the effect of Anode Hole Injection (AHI) can here be neglected, especially since the biases applied on the Control Gate (and thus on the Floating Gate) are low. Moreover, the barrier height seen by holes from the substrate is higher than the one seen by electrons from the Floating Gate (4.8 eV and 3.1 eV respectively), thus considerably reducing their injection probability.

### Experimental results

**Figure 3:**
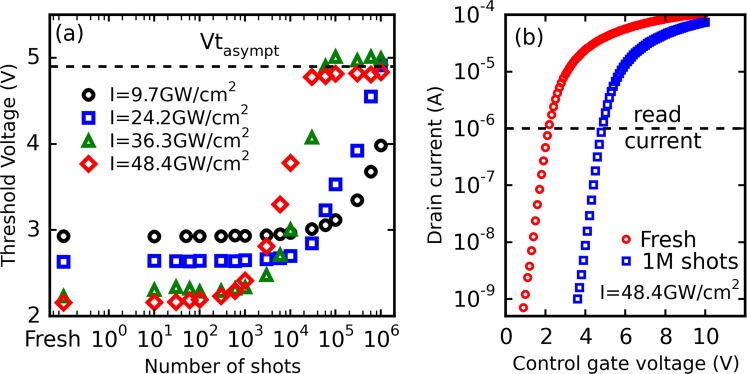
(**a**) Evolution of experimental *Vt* of erased cells as a function of the number of laser shots at different intensities (*V*_*CG*_ = 0 V). (**b**) Evolution of the drain current as a function of the control gate voltage before and after 1 million laser shots at 48.4 GW/cm^2^.

In our experiments, the laser pulse energy was adjusted but ultimately, the intensity *I* is the parameter enabling the direct evaluation of the electron density *n*_*e*_ during laser irradiation. Due to the extremely low absorption coefficient at 1300-nm wavelength (*α* = 4.5 × 10^−5^ cm^−1^), the intensity transmitted through 1 mm of silicon is >99.9% of the input intensity, according to Beer-Lambert law^27^. Therefore, the absorption of the material at this wavelength is negligible. The associated transmission coefficient can thus be calculated using Fresnel equations at normal incidence and the intensity in the vicinity of the cell can be easily obtained by measuring the intensity in air and multiplying it by the transmission coefficient *T* = 69.1%. Nonlinear propagation phenomena could theoretically limit the energy deposition in the vicinity of the cell. However, due to the extremely low energy values (a few hundreds of pJ) employed in our study, these phenomena have been shown to be negligible^3^. A typical experimental acquisition of the threshold voltage *Vt* as a function of the number of laser shots for initially erased cells at *V*_*CG*_ = 0 is displayed in Fig. 3a. These results show that the erased cells are gradually programmed after cumulative laser shots as *Vt* evolves according to a “S-shaped” curve within this semi-logarithmic plot. One can note that the starting point is different for each dataset due the different erased threshold voltage values (between 2 and 3 V) of the tested cells. While the asymptotic threshold voltage *Vt*_*asympt*_ remains the same in each case, it can be observed that increasing the laser pulse intensity *I* accelerates the evolution of *Vt*, which reaches its final value after fewer laser shots. This is consistent with the dependence of the number of laser-produced free carriers with two-photon absorption. Drain current characteristics retrieved during *reading* operations before laser irradiation and after 10^6^ laser shots (*I* = 48.4 GW/cm^2^) are shown in Fig. 3b. The measured *Id*(*V*_*CG*_) characteristic is merely shifted to the right under laser irradiation and is not subject to deformations that are often taken as experimental evidence of device degradation, whether resulting from electrical stress or laser-related deterioration^18,28^. Further experiments show that this result is valid for laser intensities up to about 110 GW/cm^2^ and that, at least in the 9.7–48.4 GW/cm^2^ range investigated in the present work, this shift can most likely be solely explained by the charging of the floating gate with charge carriers transiting through the tunnel oxide, which is compatible with the model described above.

### Modelling the threshold voltage evolution with the number of laser shots

**Figure 4:**
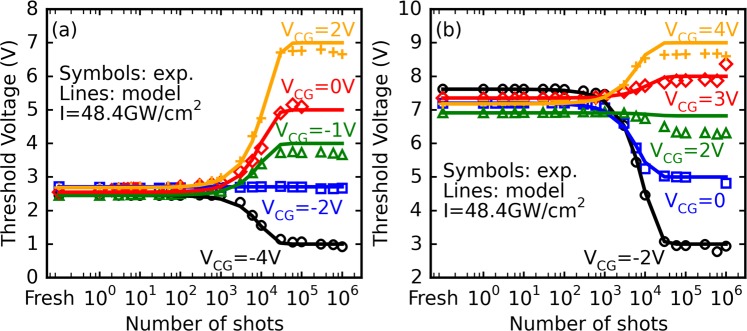
Evolution of *Vt* of (**a**) initially erased and (**b**) initially programmed memory cells, as a function of the number of laser pulses for different control gate biases. The laser pulse intensity is set to 48.4 GW/cm^2^.

The experiment described in the above section has then been carried out (*I* = 48.4 GW/cm^2^) for both initially erased and programmed Flash cells biased by several control gate voltage values. The resulting “S-shaped” curves, displayed in Fig. 4 (markers), show that the asymptotic threshold voltage differs according to the *V*_*CG*_ value applied to the device. In particular, the application of a sufficiently negative *V*_*CG*_ value on initially erased cells leads to a threshold voltage evolution contrary to the one monitored in the *V*_*CG*_ = 0 V case (and vice-versa for initially programmed cells). The experimental “S-shaped” curves, can be modelled using a generalized sigmoid function of the following form:

2 $${V}_{t}(n)=A+\frac{B}{1+{e}^{-Cn}}$$

where *A*, *B* and *C* are three fitting constants, and *n* the number of applied laser shots.

Knowing the two extremal threshold voltage values *Vt*_*start*_ and *Vt*_*asympt*_ (i.e., when *n* = 0 and *n* → ∞ in the equation above) through electrical characterization, expressions for *A* and *B* can be derived by solving a two-equation system, and Eq. (2) finally becomes:

3 $$Vt(n)=2V{t}_{start}-V{t}_{asympt}({V}_{CG})+\frac{2(V{t}_{asympt}({V}_{CG})-V{t}_{start})}{1+{e}^{-Cn}}$$

The fitting curves (full lines) obtained with this model for erased and programmed cells are consistent with the experimental results displayed in Fig. 4 for a well-chosen value of parameter *C* which is independent of the applied control gate voltage (*C* = 1.5 × 10^−4^ ± 0.5 × 10^−4^ for all the curves). These results thus demonstrate that, when the laser intensity is low enough to avoid device degradation, it is possible to limit or enhance the laser effect by applying a suitable control gate bias and that the resulting evolution of the threshold voltage can be predicted by relatively simple means.

**Figure 5:**
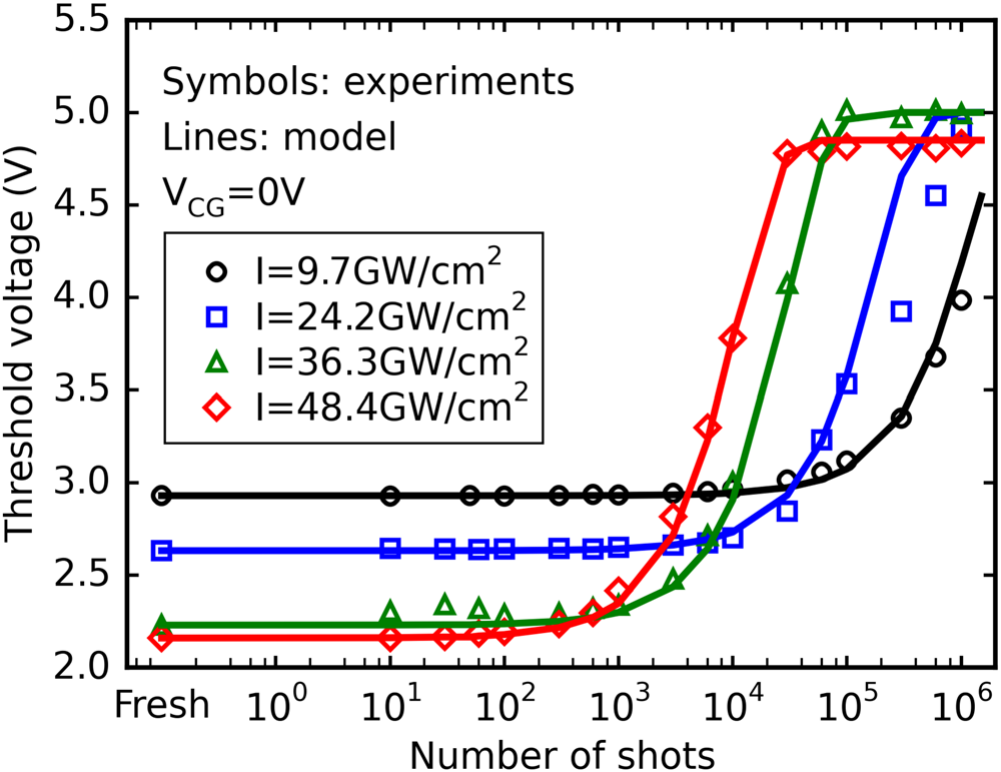
Evolution of the threshold voltage shift of initially erased cells as a function of the number of laser shots at different intensities and *V*_*CG*_ = 0 V.

**Figure 6:**
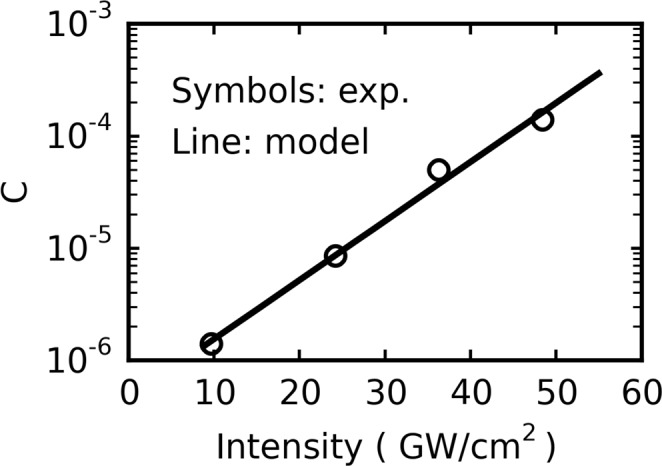
Graphic representation of the function log(*C*) = f(*I*) obtained from the *C* and *I* values listed in Table 1.

The model has then been used to fit the set of data previously displayed in Fig. 3a and reproduced in Fig. 5. The values of the growth rate *C* used to reproduce the experimental curves are listed in Table 1 and displayed in Fig. 6 as a function of *I*. It can be observed that *C* exponentially increases with laser intensity in the 9.7–48.4 GW/cm^2^ range, and can thus be written as:

4 $$C\approx {C}_{0}\,{\exp }(\frac{I}{{I}_{0}})$$

with *C*_0_ = 4.6 × 10^−7^ and *I*_0_ = 8.2 GW/cm^2^ as extracted from Fig. 6. The fact that *C* does not vary as *I*^2^, as could be expected for a mechanism induced by two-photon ionization, shows that the number of free carriers cannot be the only explanation for the observed evolution of the threshold voltage under laser irradiation, but that their intensity distributions should play a large role as well.

**Table 1:** Value of parameter *C* for different pulse intensities (*V*_*CG*_ = 0 V).

Laser intensity	9.7 GW/cm^2^	24.2 GW/cm^2^	36.3 GW/cm^2^	48.4 GW/cm^2^
*C*	1.4 × 10^−6^	8.5 × 10^−6^	5.0 × 10^−5^	1.4 × 10^−4^

**Figure 7:**
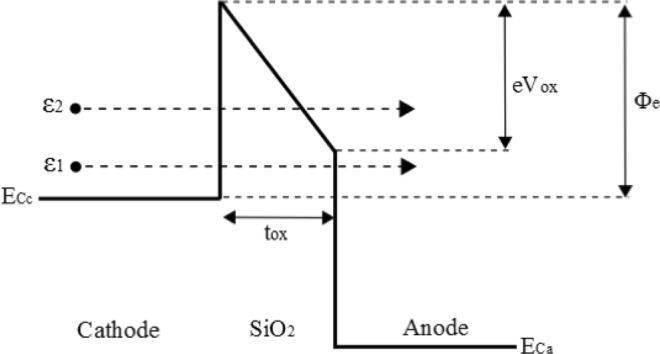
Upper part of the band diagram of the tunnel capacitor. According to the sign of *V*_*ox*_ (and thus the value of *V*_*CG*_), the role of the cathode can be either played by the floating gate (*V*_*ox*_ < 0) or the substrate (*V*_*ox*_ > 0). Electrons with energies of *ε*_1_ and *ε*_2_ with respect to the bottom of the cathode conduction band *E*_*Cc*_ will respectively meet a trapezoidal and triangular tunneling barrier. Lower part of the band diagram is omitted as tunneling of laser-generated free holes should hardly contribute to the variation of *Q*_*FG*_ due to the discrepancy between Si-SiO_2_ barrier heights for electrons and holes (*ϕ*_*e*_ = 3.1 eV, *ϕ*_*h*_ = 4.8 eV).

An increase in the deposited energy per laser pulse will likely induce higher densities of laser-generated carriers that are potential candidates to transit through the tunnel oxide^3^. Moreover, carriers created by two-photon ionization, which remains the main electron generation process at 1300 nm in silicon in the femtosecond regime, can subsequently absorb additional photons via an inverse Bremsstrahlung process^29,30^ as long as the laser field is present (during the pulse). This should result in generally more ‘heated’ energy distributions for the carriers, and thus a higher tunneling probability as the transmission coefficient for both electrons and holes can be expressed, thanks to the commonly used Wentzel–Kramers–Brillouin (WKB) approximation, as^31–34^:

5 $${T}_{triangle}(\varepsilon )={\exp }\{-4\frac{\sqrt{2{m}_{oxe,h}^{\ast }}}{3\bar{h}e{E}_{ox}}{({\varphi }_{e,h}-\varepsilon )}^{\frac{3}{2}}\}$$

6 $${T}_{trapeze}(\varepsilon )={\exp }\{-4\frac{\sqrt{2{m}_{oxe,h}^{\ast }}}{3\bar{h}e{E}_{ox}}[{({\varphi }_{e,h}-\varepsilon )}^{\frac{3}{2}}-{({\varphi }_{e,h}-e{E}_{ox}{t}_{ox}-\varepsilon )}^{\frac{3}{2}}]\}$$

where ${m}_{oxe,h}^{\ast }$ and *ϕ*_*e*,*h*_ are respectively the SiO_2_ effective mass and the Si-SiO_2_ barrier height for electrons and holes tunneling through the oxide, *E*_*ox*_ is the norm of the oxide electric field, and *ε* the free-carrier energy with respect to the bottom of the conduction band (electrons) or the top of the valence band (holes). According to its energy, a laser-generated carrier will either face a triangular or trapezoidal barrier, as seen in Fig. 7, which explains the need for two separate transmission coefficient expressions. The oxide electric field, for a tunnel capacitor submitted to a *V*_*FG*_ − *V*_*B*_ potential difference, can be written as^35^:

7 $${E}_{ox}=\frac{|{V}_{ox}|}{{t}_{ox}}=\frac{|({V}_{FG}-{V}_{B})-{V}_{FB}-{\psi }_{S}+{\psi }_{FG}|}{{t}_{ox}}$$

where *V*_*FB*_ is the flatband voltage of the capacitor, *t*_*ox*_ its oxide thickness, *ψ*_*S*_ and *ψ*_*FG*_ the surface potentials of the substrate and floating-gate respectively.

Assuming a density of generated free electron-hole pairs in the 10^19^ cm^−3^ range in both electrodes for the investigated laser intensities^3^, and assuming that a part of these carriers will be removed from each of the space charge layers due to the reigning electric field, a carrier surplus will exist in each electrode compared to the thermodynamic equilibrium case. Under these conditions and the reasonnable approximation of a metallic behaviour for both electrodes, the expression of the oxide electric field merely reduces to:

8 $${E}_{ox}\approx \frac{|({V}_{FG}-{V}_{B})-{V}_{FB}|}{{t}_{ox}}$$

This last equation, when combined with Eqs (5) and (6), allows a deeper understanding of the experimental results displayed in Figs 4 and 5. At a given laser intensity, and considering the fact that |*V*_*FG*_| = *f*(|*V*_*CG*_|) is an increasing function (see supplementary material of our previous work^19^), the oxide electric field and thus the transmission coefficient of tunneling electrons will be enhanced as |*V*_*CG*_| gets higher. These considerations should explain why, as seen in Fig. 3 for both initially programmed and erased cells, a given threshold voltage value can be reached “faster” (i.e. a given amount of floating gate charge variation is achieved through fewer laser shots) for higher |*V*_*CG*_| values. Conversely, Fig. 5 shows the asymptotic threshold voltage can be reached faster, for a given *V*_*CG*_ value, when the laser intensity is increased. This acceleration can most likely be explained by the fact that, as previously stated, an increase in *I* (and thus in energy) should lead to a general increase in *ε* and transmission coefficient for the laser-generated carriers. Looking back at the simulation results displayed in Table 1, this likely corresponds to a suitable physical explanation for the laser intensity dependence of the coefficient *C* introduced in Eq. (2).

### Cancelling the laser effect on the flash cells and analytical derivation of the asymptotic voltage

**Figure 8:**
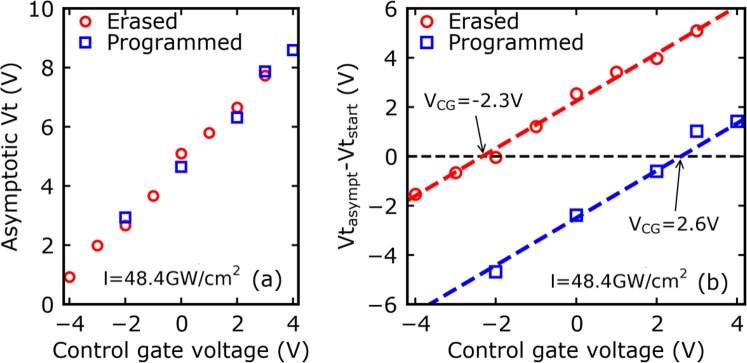
Evolution of (**a**) the asymptotic *Vt* and (**b**) the shift *Vt*_*asympt*_ – *Vt*_*start*_ as a function of the CG biasing voltage (*I* = 48.4 GW/cm^2^) for initially erased and initially programmed cells.

Previously in Fig. 4, it can be observed that for a certain control gate voltage the effect of the laser irradiation on the evolution of the threshold voltage can be cancelled. According to these measurements, this particular *V*_*CG*_ value, for *Vt* to remain constant should respectively be around −2 V and in the +2 V to +3 V range for initially erased and programmed cells. To investigate this matter, asymptotic values of the threshold voltage are shown in Fig. 8 as a function of the control gate voltage. Results displayed in Fig. 8a show that *Vt*_*asympt*_ follows a linear dependence with *V*_*CG*_ with a slope equal to unity, so the asymptotic voltage can be expressed as:

9 $$V{t}_{asympt}=a{V}_{CG}+b={V}_{CG}+b$$

where *a* and *b* are constants.

**Figure 9:**
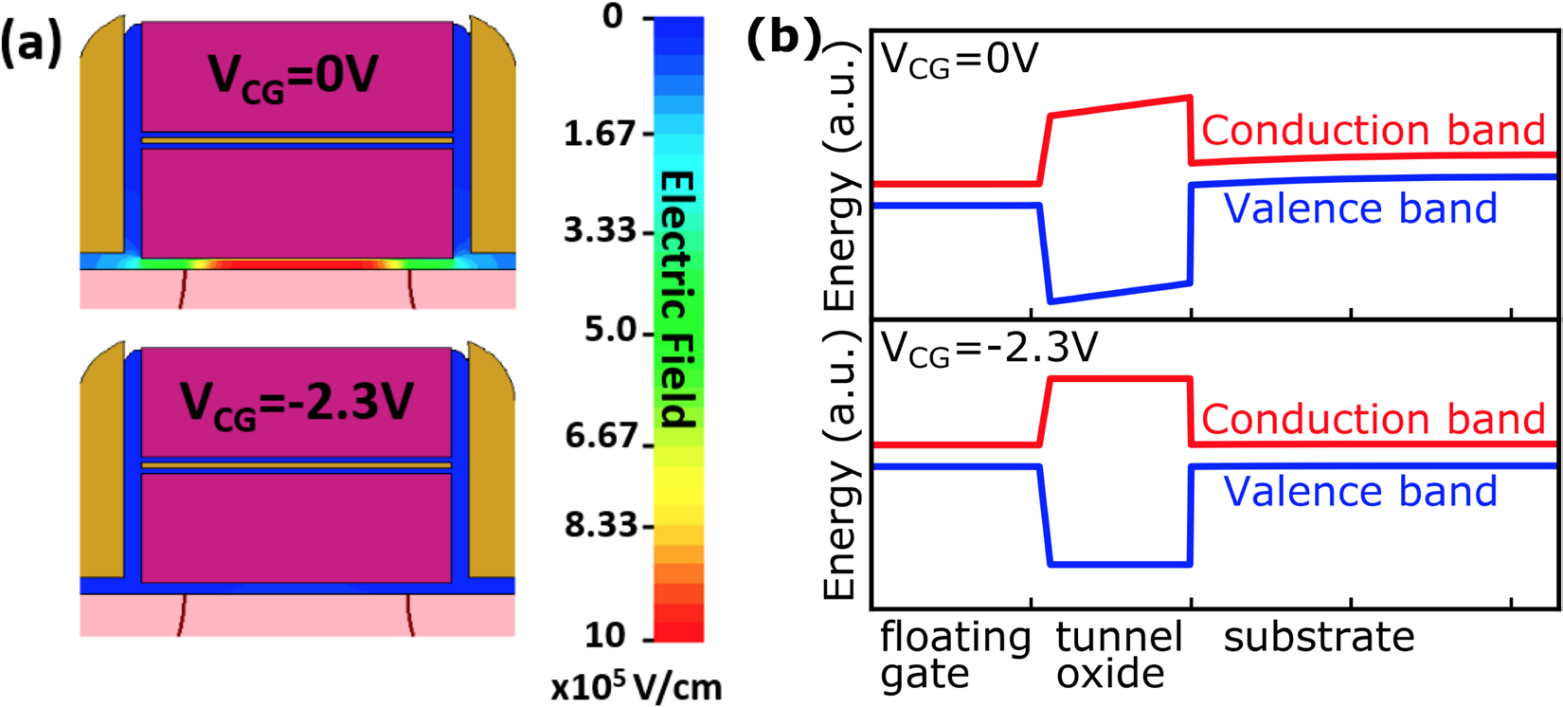
(**a**) Distribution of the electric field across the tunnel oxide of an erased device and (**b**) band diagrams of the injection zone obtained with TCAD simulation for different biasing conditions (*V*_*CG*_ = 0 V and *V*_*CG*_ = −2.3 V).

Moreover, one can observe that for a given *V*_*CG*_, laser irradiation leads to the same *Vt*_*asympt*_ value for each memory cell, whatever its initial state. In other words, coefficients *a* and *b* should not depend on the initial quantity of charge stored in the Flash cell. According to Fig. 8b, the needed *V*_*CG*_ values to cancel the effect of laser irradiation in initially erased and programmed cells (i.e. *Vt*_*asympt*_ − *Vt*_*start*_ = 0) are respectively −2.3 V and +2.6 V, which is coherent with the above predictions. The physical significance of these two values has then been investigated with the help of Technology Computer Aided Design (TCAD) simulations whose results are displayed in Fig. 9.

The simulation of the electrical behaviour of the initially erased and programmed Flash cells show that, in both cases, the band diagram of the tunnel capacitor can be “flattened” when the *V*_*CG*_ values extracted from Fig. 8b are applied on the control gate. The threshold voltage remains constant because no net charge transit is possible through the tunnel oxide as the band diagram is flat, in accordance with^19^. Under the hypothesis that the *Vt*_*asympt*_ is reached when the band diagram of the tunnel capacitor becomes flat, which has been demonstrated through the results presented in this section of the report, and considering the drain, source and bulk terminals to be grounded (*V*_*D*_ = *V*_*S*_ = *V*_*B*_ = 0 V), we were able to derive in one of our previous works^19^ the following relationship:

10 $$V{t}_{asympt}=V{t}_{0}-\frac{{V}_{FB}}{K}+{V}_{CG}$$

where *K* is a constant dependent on the Flash cell geometry (*K* ≈ *0.7* for the devices tested here).

This last equation is totally coherent with Eq. 8 and the experimental results of Fig. 8, and the last remaining parameter can thus be identified:

11 $$b=V{t}_{0}-\frac{{V}_{FB}}{K}$$

Finally, the combination of Eqs (3) and (11) yields the following expression for the threshold voltage:

12 $$Vt\,(n)=2V{t}_{start}-{V}_{CG}-(V{t}_{0}-\frac{{V}_{FB}}{K})+\frac{2[{V}_{CG}+(V{t}_{0}-\frac{{V}_{FB}}{K})-V{t}_{start}]}{1+{e}^{-Cn}}$$

Knowing the laser intensity dependence of *C* and since all the other physical quantities can be determined or set (*V*_*CG*_), the threshold voltage of the irradiated Flash cell can thus be predicted after any number of laser shots thanks to this last equation, provided that the laser intensity is low enough to avoid device degradation.

## Conclusion

In this study, the behaviour of Flash memory devices under backside irradiation with infrared femtosecond laser pulses has been investigated. Drain current characteristics, measured before and after laser irradiation, have shown that within a limited laser intensity range, the progressive shift of the threshold voltage was not linked to any kind of device degradation and could most likely be solely explained by transport of carriers through the tunnel oxide. Further experiments involving control gate biasing and variation in laser intensity proved that this effect can either be enhanced or limited, and that, under particular circumstances, it could be suppressed altogether. These results should thus show plenty of applications in microelectronic device reliability as well as in ultrafast microelectronics. Moreover, a predictive phenomenological model, supported by TCAD simulations, has been developed to describe the threshold voltage evolution regardless of the initial state of the cell, the laser intensity and the biasing voltage. Further understanding could probably be achieved in the future through time-dependent modelling of the different physical phenomena involved during the laser pulses (i.e. interaction of charge carriers with laser-generated photons as well as with the silicon lattice^35–38^ through Monte-Carlo simulations). It would allow, in particular, the calculation of the energy distribution of laser-generated carriers which plays an essential role in the tunnel transit of the latter, as the strong dependence of the growth parameter *C* with laser intensity has likely shown.

## Materials and Methods

### Tested samples

Electrical measurements have been performed on single isolated Flash cells (tunneling area ~0.015 µm²) that are embedded on a 200 mm wafer. The thicknesses of the tunnel oxide and the Oxide/Nitride/Oxide stack are approximately 10 nm and 15 nm respectively. The source and drain regions are both in n-type, while the substrate is p-type (doping concentration ~10^18^ cm^−3^). The thickness of the c-Si substrate is 1 mm.

### Experimental setup

The experimental setup employed to irradiate the cells from the polished rear surface of the silicon wafer is detailed elsewhere^18^. Briefly, it relies on 100 fs pulses emitted by a Titanium-Sapphire laser source at 1 kHz repetition rate. The wavelength is converted from 800 nm to 1300 nm (photon energy, 0.95 eV) thanks to an Optical Parametric Amplifier for reaching the transparency domain of silicon. The pulse energy (and thus the intensity) is adjusted optically by combining a half-wave plate and a polarizer. The beam is focused by an objective lens of numerical aperture 0.42. At the focus, the beam is Gaussian-shaped with a diameter at 1/e^2^ of about 2 µm, and the Rayleigh length in silicon is around 8.2 µm, i.e., much larger than the total thickness of the irradiated device (i.e. the stack of layers in the memory cell, shown in Fig. 1a). Therefore, the spatial distribution in intensity can be considered as uniform during its propagation through the Flash memory cell.

### Data acquisition and processing

Electrical measurements and the various associated operations were performed using an Agilent 4156 C semiconductor parameter analyser^39^:

#### Reading operation

Drain current characteristics of the tested Flash cells (i.e. *Id*(*V*_*CG*_) curves) are retrieved after a given number of applied laser shots. As showcased in Fig. 2b, the threshold voltage has been measured throughout this study as the control gate voltage for which the drain current is equal to a reference current specific to the Flash technology used (1 µA in this case).

#### Programing/Erasing operations

Flash cells are typically programmed via hot-carrier injection while they are erased using Fowler-Nordheim conduction. Both operations are generally carried out on an independent experimental setup allowing the application of well-defined electrical pulses (amplitude, plateau, rise and fall times) on the cell terminals thanks to pulse generators^40^. In order to avoid any further complexification of the setup used in this study, pulse generators were not used here and both operations were realized applying a single time-limited control gate signal of suitable amplitude. The duration and amplitude of these signals were chosen so as the threshold voltages associated to prog/erase operations were consistent with their standard values (about +2.5 V and +7.5 V as seen in Fig. 3). Because of small fabrication differences between the different Flash cells tested during this study (dispersion), the starting threshold voltage associated to each operation is not identical for every device^41,42^.

#### Laser irradiation under biasing voltage

The biasing of the control gate was realized using the “sampling” mode of the 4156 C parameter analyser. The duration of the control gate signal was determined by the number of applied laser pulses between two consecutive threshold voltage measurements and the 1 kHz repetition rate of the laser setup.

### TCAD (Technology Computer Aided Design) simulations

The electrical behaviour of the tested Flash cells has been simulated from model files accounting for their fabrication process with the help of Sentaurus^43,44^ TCAD suite. Generally speaking, the software numerically solves physical equations, such as diffusion and transport equations, to model the structural properties and electrical behaviour of semiconductor devices. In order to obtain the results displayed in Fig. 6, the model was first calibrated by tweaking a few process parameters so that the threshold voltages extracted after simulating the programing and erasing operations coincide with the measured ones. Band diagrams of initially programmed and erased cells were then calculated for respective applied control gate voltages of +2.6 V and −2.3 V.

## Data Availability

The datasets generated during and/or analysed during the current study are available from the corresponding author on reasonable request.
